# Mapping key issues and useful theory to address remaining ethical, practical and political challenges in participatory research in health

**DOI:** 10.12688/wellcomeopenres.24114.1

**Published:** 2025-08-04

**Authors:** Robin Vincent, Janet Harris, Rene Loewenson, Helene-Mari Van der Westhuizen, Gill Black, Geoff Wong, Sassy Molyneux

**Affiliations:** 1 Centre for Tropical Medicine and Global Health, Nuffield Deparment of ClinicalMedicine, University of Oxford, Oxford, OX3 7LG, UK, UK; 2 Robin Vincent Learning and Evaluation Ltd, Sheffield, S89FH, UK; 3 Division of Population Health, School of Health and Related Research ScHARR, University of Sheffield, Sheffield, Sheffield S1 4DA, UK; 4 TARSC, Harare, Zimbabwe; 5 Sustainable Livelihoods Foundation, Cape Town, South Africa; 6 Nuffield Department of Primary Health Care Services, University of Oxford, Oxfrod, OX2 6GG, UK; 7 7. Kenya Medical Research Institute (KEMRI) Wellcome Trust Research Programme, University of Oxford, Kilifi, 80108, Kenya

**Keywords:** Participation, participatory action research, participatory research, power, lived experience, co-production, knowledge generation, realist review

## Abstract

Participation is recognised as having a key role in health, for increasing the relevance and effectiveness of health interventions, for the health promoting benefits of community empowerment and as an ethical imperative. Participatory approaches to health research are also increasingly valued for bringing the insights of lived experience, and more relevant research and action. In this paper, we explore key remaining issues in participatory action research highlighted by scholars, practitioners and published literature, and highlight some useful conceptual resources which help to better understand them. We distinguish participatory action research as a paradigm involving those most affected throughout the research process, contrasting it with the more limited use of participatory tools and methods. We outline several aspects of participatory action research in health that would benefit from further theoretical and practical development, including: shifting power in the research process; the compatibility of participatory research with biomedical research; linking local inquiry and action to broader changes in policy and practice; and working with experiential knowledge in a rigorous research process. We highlight useful theory from a range of disciplines (including beyond the participatory research literature) that helps to understand some of the key processes and dynamics implicated in the issues highlighted and how this affects the outcomes achieved. We outline and share these conceptual/theoretical resources, identified as part of preparation for conducting a realist review on participatory action research in health, to contribute to ongoing reflection and development in the field.

## Plain language summary

Participation of community members and members of the public in the design of health interventions can increase their relevance and effectiveness. Such participation can also empower people, which has its own health benefits. Participation is also a good in its own right for the way it respects the value of involving the full range of people in any activity. Participatory approaches to health research are also increasingly valued for drawing on the insights of people’s lived experience, to lead to more relevant research and action. In this paper, we explore key remaining issues in participatory action research highlighted by academics, those involved in practical projects and in published literature. We also pull out some of the key ideas that have helped to better understand how participation works in practice. We distinguish ‘participatory action research’ as an approach to research that involves the people most affected by an issue throughout the research process, and contrast this with more limited use of participatory tools and methods at only one stage or another. Several aspects of participatory action research in health that could be better understood include: shifting power in the research process; how easily participatory research can be combined with biomedical research; whether things learned in participatory action research lead to broader changes in health services and policies; and how research can draw on peoples lived experiences in a balanced way. We highlight some useful ideas from other fields to help illuminate participatory action research in health. The paper is the first stage of a more comprehensive review our research team are conducting over the next two years and provides a summary of current ideas to contribute to ongoing discussions in the field of participatory action research in health.

## Introduction

Participation is recognised as having a key role in health, for increasing the relevance of health interventions, for the health promoting benefits of community empowerment, and as an ethical imperative, although the history of its application in practice has been uneven ^1^. Participation in health research is also increasingly seen as valuable, for generating insights from lived experience, and to ensure research is ethical, and relevant for addressing real-world challenges ^2, 3^. The value of such approaches has been strengthened by contributions from complexity and systems thinking and developments in social theory, which suggest that complex social issues such as health and wellbeing demand methods that are able to draw on and systematise the experiences and analyses of people most affected ^4, 5^. The prevailing health paradigm, which emphasises curative interventions over addressing the social determinants of health ^1^, may present particular challenges and opportunities for participatory enquiry, and its ability to involve those most affected by health issues.

Participatory Research (PR) or Participatory Action research (PAR) can be broadly understood as research ‘where people affected by the issue being studied are involved ***throughout*** the research process’ (3; our emphasis). Cycles of action and reflection coupled with collective analysis are central to the research method – more commonly given specific emphasis in Participatory Action Research PAR ^6, 7^. PAR/PR has grown into a mature field that proponents argue is able to rigorously address the challenges of researching complex social phenomena. We distinguish PAR and PR that emphasises action as integral (referred to as PAR from now on in this paper) from a range of applications of participatory tools and methods confined to particular stages in the research process (see below). Many early methodological criticisms of PAR are being constructively engaged with, including questions around objectivity, risks of co-optation of participants, attention to gender and other differences, and an underpinning by western assumptions ^7^.

Maturing theory and practice in PAR have highlighted several key issues where further analysis may support a better understanding of the social dynamics, paradigms, and methods involved, and the power relations at stake. In this paper, we outline some of these key areas of PAR and consider some of their characteristics and implications.

These key areas are:

1. The benefits and challenges of shifting power at a range of different levels in the research process and the potential complementarity of PAR and predominant models of scientific research, including the risk of co-optation.
2. Working with ‘experiential knowledge’ and participatory analysis in research and reconciling diverse ways of knowing in a rigorous inquiry process.
3. The possibility and challenge of linking local inquiry and action to broader changes in social life, policy and practice, particularly by affected groups.

**Figure 1.**
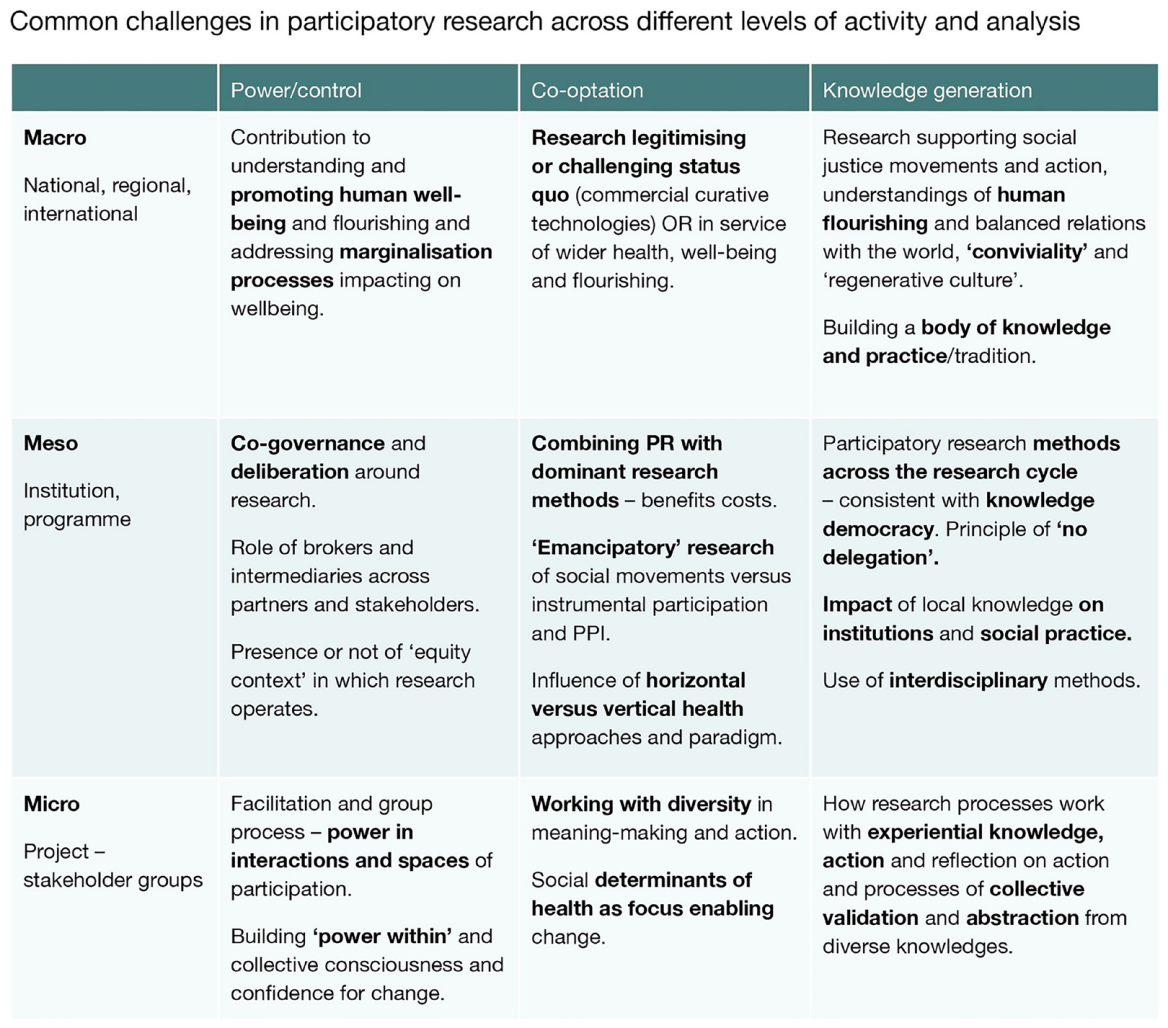
Key challenges across dimensions of participatory research (Source: the authors). Common challenges in participatory research across different levels of activity and analysis.

These issues are inevitably interwoven and play out at several levels. The matrix in Figure 1 summarises key issues of power and control, co-optation, and knowledge generation across micro, meso and macro levels and some of the overlaps between them. We unpack a range of these issues in the paper.

### Realist analysis of participatory research processes

Realist analysis is increasingly recognised as valuable for unpacking complex social challenges, by highlighting some of the causal processes that underpin them and how these interact to produce the pattern of outcomes observed ^8– 10^. As a socially negotiated process, PAR necessarily involves social and psychological dynamics at a range of scales – as reflected in the issues outlined above. Such dynamics include; psychological processes of cognition, meaning making and identity; group dialogue and interactions; organisational partnership and governance processes; and the influence of wider social, political and economic arrangements. All of these mutually affect one another, and issues of power are played out at every level. Realist analysis recognises that each of these levels may have its own distinctive identifiable causal dynamics, but that these interact with those at other levels to produce overall social outcomes ^11^. In this paper, using such a realist lens, we highlight some conceptual resources that may help to better understand some of the social and psychological processes at different levels, important contextual influences, and the linkages and interactions between them.

### Consultation and scoping for a realist review of participatory action research in health

We are conducting a Wellcome-funded realist review of PAR in health, called REAL2. The scoping phase for the main review involved engaging with contemporary scholars and practitioners using PAR methods across a range of fields and contexts, in a variety of one-to-one consultations and facilitated discussions, exploring developments in the PAR literature, and an extended ‘social theory gleaning’ process to help develop initial causal accounts of the relational dynamics identified as key to the PAR process. Conceptual resources, case studies and literature highlighted by those consulted were drawn on to iteratively refine our initial programme theory outlining ideas about how PAR works, for whom and in what circumstances. We incorporated the authors own wide experiences in a range of fields in the global north and south, including community development, participatory communication in international development, health systems and public health, engagement with health research, labour and political movements, participatory evaluation and PAR.

In this paper we share the conceptual resources and the set of working hypotheses gathered in our initial programme theory (presented towards the end of the paper) to contribute to ongoing reflection and both conceptual and methodological development in the field of PAR. We recognise that this paper represents an inevitably partial drawing together of suggestive lines of inquiry that will need further refinement in our realist review process. However, engagement with scholar/practitioners, advisors and funders suggest that the theoretical resources and initial programme theory are a valuable resource in their own right.

### Power and control: benefits and challenges of power sharing in the research process

Since the initial development of participatory approaches as part of Latin American social justice movements in the 1970s ^12, 13^, and separately, as part of community organizing in the Global North ^14^, methods and tools of participation have been used across diverse settings, with an associated growth of terminology. Several typologies have attempted critically to characterise the degree of participation in particular cases, an early example being Arnstein‘s ‘ladder of participation’ which contrasts tokenistic informing and consultation at one end of the spectrum, to control by participants at the other ^15^.

Participation of people most affected by the issues being addressed throughout the research process – the citizen partnership and control pole of Arnstein’s ‘ladder’ - is considered a defining feature of PAR as a research paradigm ^2, 3, 6, 16^. Key elements of this overall PAR paradigm include the validation of experience, critical reflection on the drivers of such experience, collective analysis and learning from actions. Together these elements can lead to changes in consciousness and increases in confidence and capacity ^6, 16^. A range of research approaches, at different times and in different places, have sought to emphasize this involvement of people affected throughout the research process. These processes include: Popular Education, Participatory Action Research (PAR), Systemic Action Research (SAR), Participatory Research (PR), Community Based Participatory Research (CBPR), Community Engaged Research (CEnR) and Indigenous research. In addition, participatory techniques have often been used in more limited ways, added on to existing research paradigms where participation may be only partial, or used at particular stages of the research process, variously labelled as co-production, patient and public involvement, community engagement, and strands of co-design and human centred design.

### How much participation? – the motivations and the dangers of co-optation

The growth of interest in participatory approaches more generally may be understood as part of a struggle between attempts to widen public participation in politics, public life and knowledge generation. This has been driven by social justice movements and the social medicine movement in particular in Latin America, echoed in the New Social Movements from the late 2060s onwards, and countered by attempts to limit and manage that involvement on the part of governments and elite interests ^16– 18^. In the UK, these developments have been linked to a perceived legitimacy crises in government and public agencies following various health and service crises in the 1980s, and the increasing privatization of services and research ^19^.

In this context, public involvement of various sorts gave the appearance of responding to public concerns, while limiting the actual public influence. Mental health and service user advocate Peter Beresford has similarly highlighted the growth of consumer-based models of participation and feedback across a range of public services, including the growth of public patient involvement as a way of individualising public concern and input ^17^. He argues that this consumer model rooted in neo-liberal ideology is inconsistent with the original impulse and rationale of participatory approaches, which is to support people to be involved in collectively defining, researching, and responding to social issues themselves. In the disability field, he argues for disability-led participatory research as an independent process, complementary to mainstream research, and taking the lived experience of disabled people seriously, something we return to below.

Drawing on participatory tools and methods at particular stages of research initiatives following other paradigms, however, may limit the value and impact of such participation. Further than this, at the extreme, it may lead to co-optation of people into dominant research agendas under the guise of seeking their contribution – something which has been a perennial concern since the birth of participatory methods in Latin American social justice movements ^13^.

Concerns around the co-optation of participatory methods have played out in the field of international development and health, where participatory methods were increasingly advocated and adopted from the late 1970s onwards by non-governmental organisations, UN agencies and the World Bank, and the boundaries between research, evaluation, service design and action have been blurred. Such concerns were reflected in debates at the turn of the millennium around whether participation in development processes represented a ‘new tyranny’ ^20, 21^. The adoption of the language of participation and application of a variety of off-the-shelf techniques in a range of fields and sectors may mask a diversity of different practices, meanings and implications of participation in both theory and practice. This makes application and evaluation of participatory methods challenging and is one of the rationales for the current review.

Diverse uses of participatory approaches have been accompanied by a proliferation of terms to describe them and an eclectic borrowing of language and methods across fields. This lack of conceptual clarity and inconsistency of practical application limits understanding of the relational and power dynamics involved in PR and the core components and contributions of PAR as a paradigm. Participation is commonly advocated as a broadly ethical good, and sometimes invoked more for the instrumental and political benefits of the apparently better knowledge of an issue gained from talking to people who have lived experience of it. In the case of international health research, ethical guidelines now mandate processes of community engagement throughout the research process ^22^. However, there is a need to move beyond these broadly declared rationales to understanding some of the dynamics of participation in particular instances, and how key mechanisms of participation may be affected by the contexts in which they take place and lead to a range of outcomes, both intended and unintended. Here, a realist logic of analysis that looks systematically at outcomes and related contexts, and relates these to the actual processes undertaken, promises to be fruitful for better understanding of participation.

### Power and participation

PAR identifies knowledge as one source of dominant power. Rather than ‘power over’ that privileges the knowledge of one group over another, PAR generates a shared ‘power with’ through collective analysis by those affected of the causes of their ill health, and a ‘power with’ to build confidence to produce change in these causes ^23, 24^. One attempt to address the uneven application of participatory methods and better analyse the spectrum of participation and the different forms of power they reflect, has been to draw on the notion of ‘spaces of participation’ ^25, 26^. This seeks to draw attention to and analyse the terms of engagement and specific practices involved in any instance of participation. The distinction between ‘invited’ spaces and ‘claimed’ or ‘created’ spaces of participation can be useful to understand the difference between people being asked to contribute to a process largely managed and designed by ‘experts’, and one where the spaces of participation and related processes are designed and led by those involved in and affected by the issue at hand. The distinction also echoes that between PAR as a paradigm and as a set of more limited tools or techniques added on to an unchanged overall research paradigm. In a similar fashion, the notion of ‘affordances’ is useful for drawing attention to how particular settings and sets of relations, may help or hinder the potential to realise participatory processes; a framing that has been usefully applied to understand the impact of digital technologies on participatory processes ^27^.

### Practices of participation

The focus on actual practices of participation and the terms on which they are undertaken is a useful way to complement some of the more general frameworks for understanding power ^28– 31^ by analysing particular instances of participation and their implications. Some strands of critical realist theory offer further conceptual resources for understanding the distinctive character of power in a range of social processes including, informal social interactions, the influence of social norms and roles, organisational processes, and the development of culture, knowledge, cognition and shared meaning ^8, 32– 36^. Each of these processes may have distinct causal dynamics, stemming from the particular interactions involved ^11^. In this way, analysis can move beyond more abstract notions of power to the concrete type of power involved in each aspect of social action, co-ordination and knowledge generation ^35^. We return to the role of power in knowledge generation below. The importance of inter-subjective dynamics and practices is also highlighted in work on the phenomenology of power ^37^. In this way there are a range of analytical tools that promise a more tangible analysis of power dynamics in participatory processes.

### Capacity for participation

It is also important to recognise the risks, time commitments and opportunity costs involved in asking people to participate in research, particularly when those people are already experiencing challenges of poverty and marginalisation. This suggests a need to link PAR to existing forms of collective organisation, and to adequate support, including emotional support and conflict resolution, as well as skills and capacity development for co-researchers. It also flags the importance of sustainable long-term engagement with people most affected by issues being researched to avoid the harm of research funding being short term or intermittent and damaging the relationships built and livelihoods developed ^10^. Previous realist reviews of the partnership dynamics fostered by community based participatory research have highlighted the importance of building equitable partnerships over time for supporting the capacity and sustainability of local organisations, and their ability to set their own research agendas ^38^. The role of funders is also important, and the relationship of external inputs to the in-kind resources and commitments of local people and organisations working to find solutions in local contexts ^39^. The undue influence that accompanies provision of funding is also a challenge, something that has been explored in international development settings where social movements seek to ensure downwards accountability to the groups they represent and insulate local action from implicit donor agendas ^40^.

### Group dynamics

In participatory research, the dynamics of small group processes and how a safe space with rapport and equity between people is created is important. A recent review suggests that dealing with power differences within groups and the role of the facilitator remain under-explored ^41^. One response to this has been to work in parallel with different groups to articulate distinctive experiences using creative methods, while building a means to share them with others to build understanding across groups over time ^7, 42^. The influence of prevailing social and cultural dynamics on the relational dynamics of groups, and conversely, the scope for intentionally designing and facilitating group processes in a way that may prefigure more equitable relationships is also another area of importance ^43^. A diversity of practices under the umbrella of ‘psychologies of liberation’ seeks to facilitate equitable dialogue processes, and use arts-based tools to share and collectively analyse experience, and in particular to enable marginalised experiences to be acknowledged, witnessed and responded to. Such practices seek to both build the confidence and hope of groups of people who have been subject to ongoing marginalisation or more specific periods of traumatic social conflict, and collaboratively work towards restorative action that promotes social justice (ibid). The dynamics of group processes and how to embrace diverse perspectives while building towards joint actions is an area that demands further exploration, drawing on a body conceptual resources that have long been undercurrents to the currently dominant cognitive-behavioural psychology.

### Co-governance of research

Another recent development, particularly in the field of Community Based Participatory Research, has been to explore mechanisms for co-governance of research ^44– 46^. This work builds on earlier work on research partnerships ^38^ to consider how practices for priority setting, methods selection, and deliberation in research can involve people most affected and be consistent with a participatory paradigm. A recent review underlines the benefits of co-governance and co-design of research and the importance of equitable partnership processes in research to promote greater health equity, while calling for more work to better understand the details of how these work in practice ^47^. In the global health field, parallel debates have sought to identify priority setting mechanisms that would allow more participation in setting research agendas ^48^.

### Section summary

The issues and conceptual resources outlined above may help analysis in a number of areas where scholars and practitioners have indicated that power relations in participatory process would benefit from greater understanding. These areas are listed below, beginning with more practical/instrumental aspects of participatory process and ending with more socio-political aspects:

•   The role and power of the facilitator(s) is vital in a participatory research process, influencing how open and collaborative group dynamics are nurtured and sustained. Some scholars and practitioners have described this as a ‘black box’.

•   The role of a range of intermediaries, including local organisations, and ‘brokers’ between different interest groups in a participatory process and the actions that stem from it, which may not be part of ‘formal’ research processes.

•   The importance of power sharing/shifting in research has been approached by some through the idea of co-governance, with procedures and deliberation around research priorities and processes.

•   The role of funding in shaping the research agenda and methods used, and the impact on relationships and existing efforts to address the issues being researched.

•   The importance of developing ‘power within’ – that is the confidence and capacity building process of participatory research for those involved, as an inherent part of and outcome of any participatory process.

•   The role of power relations in general marginalisation processes that underpin the poverty, precarity and vulnerability, which systematically prevent the possibility of participation or compel others to participate.

## Knowledge generation, scientific process and the role of experiential knowledge

One distinctive component of PAR is its emphasis on reflection on the experience and action by people affected by issues as a central part of the knowledge generation process. A principle of ‘no delegation’ ^23^ sees people affected taking on all aspects of the research process themselves, and crucially being involved in the analysis of experience and data and learning from action ^49^. This has often been complemented by a body of tools to support reflection and analysis of experience and an emphasis on multiple ways of knowing and sense making, drawing on visual methods and not relying exclusively on the written word ^50– 53^. The importance of emotional responses and articulating previously unrecognised patterns of social life that may need challenging is also recognised ^54, 55^. There is a dual rationale for drawing on these broader ways of knowing: first, to work with the range of people’s embodied experience as holding insights that may be beyond easy expression in language or conventions of what counts as knowledge or reasoned argument; and second, because such methods may be more accessible, supporting a wider range of people to reflect on the world around them compared with the more ‘expert’ skills and procedures of dominant research paradigms carried out exclusively by researchers.

Tim Ingold makes the case that artistic production in many majority world cultures aims to connect people with a complex experience, rather than summarise or stand in for it; artistic practice draws people into the detail of an experience, including sensory and embodied experience, rather than attempting to provide a more abstract, distant representation of it ^56^. A similar insight informs the turn to artistic process for supporting more diverse forms of sense making ^43, 53^ in PAR, and may be useful for deepening an understanding of what it means to adequately capture the reality of experience.

### The action learning cycle and the value of experience

PAR, at its core, draws on principles of learning - the ‘action learning cycle’ that many will recognise from organizational and educational settings ^57, 58^, as well as the scientific process itself. This cycle is essentially a dialogue between empirical reality and experience, attempting to understand and frame that reality with adequate theories that not only give a good account of that reality but also enable relevant action in the world. Recognising the complex realities of issues like health, poverty and discrimination, PAR processes begin with people’s lived realities and support people to reflect and analyse their own experience to understand how it is shaped by a range of personal interpersonal, social, cultural, political and environmental forces. For PAR this process of action and analysis is undertaken by people affected by an issue, not delegated to others, including researchers.

Collective dialogue and analysis can generate meanings and understandings that move beyond received wisdom and dominant ideology to articulate a range of issues that need to be addressed, some of which are amenable to local action ^41; 43^. Realist informed cognitive science has highlighted the importance of embodied experience and the way this comes to structure both everyday understandings and meanings as well as more abstract concepts and ideas ^36^. Such work highlights that there may be limits to any voluntaristic change in such understandings. Sustained group dialogue over time may be key to unpicking some of the dominant framings and understandings of issues and working with a range of diverse perspectives in a creative process of generating new meanings ^59^, This generative process of creating new meanings is something that is characteristic of the way social movements can develop new language and understandings over time ^60^ as we return to below. Additional conceptual resources come from work on the evolution of human thinking which highlights the crucial role of collaborative action and joint attention focused on practice as underpinning processes of meaning-making and social co-ordination ^61, 62^.

Further, local action and subsequent reflection on responses to that action can provide a deeper understanding of the wider forces underpinning a particular challenge and holding it in place over time ^63^ (something that is also supported by recent developments in systems approaches – see below). This, in turn, can build motivation for collective action and efforts to influence a range of institutions, such that local action can lead to wider social change. While PAR seeks to find solutions to intractable social problems by building on the insights of people, the process also strengthens people’s capacity and confidence to analyse and act in a virtuous cycle.

PAR thus brings the ethics of who does research for what purposes to centre stage. It is no accident that PAR sees research as having a dual purpose of supporting knowledge production and action - recognising the two are intimately related in human practice. It is driven by the overriding concern to serve the cause of social justice and the broadest human flourishing, and to strengthen the confidence to produce self-determined change.

### Challenging a narrow view of scientific process and what counts as valid knowledge

An enduring challenge for PAR is the perception that it may lack rigour or be unscientific – as part of long-standing debates around what counts as valid knowledge. PAR has tended to be associated with qualitative research in debates within the academy over the value of different scientific research paradigms, and the hierarchy of method that sees quantitative methods as superior to qualitative methods ^64^. PAR represents a different paradigm however, given its insistence on bringing action and analysis closer together and reducing the dominance of researchers over the process of inquiry ^6^.

### The politics of ‘science’

Developments and debates around research methods can also be set in wider debates situating scientific practice in a wider historical, political and economic context. Feminist and post-colonial critiques of the scientific emphasis on ‘objectivity’ have argued that rather than a neutral objectivity, mainstream science may enshrine a particular set of powerful social interests ^65, 66^. Mainstream science has tended to draw on and reinforce a historically specific set of elite, ‘western’ values – a notion that has recently resurfaced in debates on the coloniality of ^67^, and decolonization of, knowledge ^68– 71^. Critiques of the influence of corporate power and finance on academic disciplines, research agendas and knowledge production, point to the ‘enclosure’ of what could otherwise be public knowledge through the deployment of intellectual property laws and the selective use of findings for profitable technological developments ^19, 72, 73^, situated in what Naomi Klein has called a broader logic of ‘extractivism” ^74^.

These critiques are echoed in work at the confluence of design, decolonization, and social movements with the notion of ‘ontological design’ ^75^, which focuses on linking local practices, values and ways of living and being in the world with wider social, economic political arrangements and relations with the natural world. Such work questions the overall ‘civilisational model’ that has been developed through the recent historic combination of capitalist economics, patriarchy, and colonialism, and argue for a conscious participatory design of alternatives that build on indigenous traditions and cultures that combine autonomy and communality. This critique also highlights an ‘ethic of care’ common to feminist and indigenous values, and a strong emphasis on the web of relationships with life and land ^76^ that differs from the narrow instrumental rationality that underpins dominant models of social development. It also highlights how some environmental movements and initiatives aim to develop social relations and practices that may prefigure alternative ways of living that are more consistent with a sustainable planetary future. This can be seen in the ‘transition towns’ movement as well as in decision-making processes and logistics/organising of direct action movements for environmental justice. Also from the field of design comes the notion of ‘designing regenerative cultures’ ^77^, a core principle of which is to encourage relationships that sustain and perpetuate well-being and flourishing across a wide range of areas of social life and relations with nature. Across such approaches, there is a concern to link the personal and political, the local and wider social setting, drawing on consistent principles that may have a different logic to the instrumental and hierarchical one embedded in neo-liberal culture and practice. Such work draws on indigenous values and cosmologies ^74^. It also draws on Illich’s critiques of institutions and the industrial application of technology, which he argues could be developed in a more decentralised and distributed way and used in support of more diverse ways of living – what he calls ‘conviviality’ ^78^.

### The politics of knowing and epistemic justice

Another related strand of debate centres on issues of ‘epistemic injustice’ – highlighting that the experience and perspectives of some types of people have been systematically undervalued – both their knowledge and their legitimacy as someone speaking ^79, 80^. In a parallel analysis, disability and mental health movements have highlighted how their experience and knowledge has been systematically ignored, arguing for the importance of ‘lived experience’ to inform research agendas and action ^17, 81^. Rather than see lived experience as a source of bias to be discounted, as it is by much traditional research, it should be valued for providing a distinctive contribution to interpreting and understanding social phenomena. This need not undermine the rigour of research processes, since there is a recognition that lived experience is socially and historically shaped ^81^. Centring lived experience also challenges the tendency for research to distance people from analysis of their own experience, whether through dominant ideological framings of issues or assumptions about what counts as valid knowledge

### Evaluative knowledge and human flourishing

While making clear that science has developed within certain prevailing political and economic arrangements, many of the above critiques seek to retain the idea of rigorous enquiry and science, while challenging its reliance on a narrow instrumental rationality. A case is made for drawing on wider ways of knowing and types of knowledge, highlighting how science has become identified with a particular hierarchy of methods and understanding of rationality ^82^. Critical Realists in particular, have sought to draw on productive aspects of feminist and post structuralist thought, including the recognition that ways of knowing and scientific enquiry process are always socially constructed, while retaining an interest in developing broadly generalisable insights and understandings about social life and some of the consistent causal processes that shape it ^34, 82^.

For some, this has also meant recognising the inherently evaluative nature of much knowledge, and the problems with attempting to claim an objectivity that takes no position on how science and knowledge production contributes to or undermines human flourishing and well-being. Capability theorists, such as Amartya Sen and Martha Nussbaum ^83, 84^ draw attention to some broadly universal human characteristics that they argue transcend culture and setting, which may provide a more useful orienting framework for social research than traditional notions of objectivity as ‘neutrality’.

In an extensive critique of social scientific method that resonates with much of the work on epistemic injustice ^82^, Andrew Sayer emphasizes the value of everyday knowledge and experience – including the practical reason that is often tacit and embodied in people’s dispositions, and contrasts this to the more abstract instrumental knowledge of social scientists. Such everyday knowledge has a relationship of concern to and involvement in the world. Sayer argues that it is ‘objective’ in the sense that it ‘pays attention to the object’ and the details of and context in which any object of concern is embedded, resonating with Ingold’s account of perception above. People’s everyday responses, including emotional ones, are ‘about something’ and their evaluative component is also guided by values and previous experience as to what will support the wellbeing of a person and others. In this way they are not ‘subjective’ in the sense of being arbitrary or unrelated to matters at hand, but are about the world, and appreciation of and action within it from a particular position. In addition, emotional responses and dispositions are not ‘unreasonable’, but provide a commentary on ongoing relations with the world and the wellbeing of ourselves and others – something contemporary neuroscientific and theoretical work on the emotions supports ^82, 85, 86^.

Such an argument does not dismiss the value of scientific inquiry but argues that it can embrace a broader range of methods and needs to question its assumptions around validity and an overemphasis on a narrow instrumental rationality. Experiential knowledge can be an important source of knowledge about how human capabilities are enabled or stifled, and as such is a valuable and important component of scientific inquiry.

### Participation and the complexity of lived experience

Further support for the value of drawing on lived experience comes from social scientific applications of complexity and systems theories. Most social challenges, from health to climate change, are increasingly recognised to be complex ‘wicked’ problems, with emergent, often unforeseen properties stemming from multiple interacting factors and feedback loops ^4, 87^. Understanding the combination of factors that impinge on the health of particular people and groups demands methods that can access both the distinctive mix of influences at various levels in any given case, and a range of cases where these influences may manifest quite differently. Importantly, such complexity involves a combination of practical characteristics of places, economies and environment, and the ideas and meanings through which people act and respond. The latter are just as ‘real’, with tangible consequences, and are important in any research process that seeks to understand and address a complex issue ^88^. As such, any social phenomenon needs to be understood as a ‘laminated system’ made up of influences at a range of levels from individual psychology to structural factors, and as such demands an interdisciplinary approach ^89^. The notion of ‘wellbeing’ itself draws attention to the wide range of factors involved in human flourishing and has in part been used to avoid the assumptions that come with the narrower notion of health ^90^.

PAR approaches are particularly suited to engaging with this complexity and finding actionable solutions that build on people’s ability to reflect on and analyse their own realities and weave them into a rigorous process of inquiry ^4, 50^. Complexity theory also provides important insights into how particular aspects of social disadvantage can interact and compound each other. This is evident in the analysis of marginalization and poverty in international development ^91^, complementing theories of intersectionality that aim to understand difference and diversity ^92, 93^.

While an emphasis on ‘lived experience’ and the recognition of experiences is a vital starting point in PAR, reflection and collective analysis is also important to understand how experiences are socially shaped by prevailing circumstances ^41^. Recent scholarship on the politics of difference highlights a tension in contemporary identity politics between emphasising singular, incommensurable experiences and analysing these as socially produced and ultimately amenable to change in ongoing struggles to challenge discrimination and inequality ^94^. We further consider the relationship between immediate experience and the wider social forces that produce it in the next section on linking local action with wider social change.

### Section summary

Drawing on the conceptual resources outlined above may help to better understand some of the contributions and challenges of participatory health research related to knowledge production including:

•   The distinctive contribution of a rigorous participatory approach to scientific inquiry rooted in lived experience, using collective validation and critical analysis to understand how experience is socially produced, and building capacities and confidence to transform in the analysis of action and change.

•   Working with diversity and diverse experiences to better address the complex character of health, and to generate ‘shared’ meanings and action.

•   Challenging prevailing assumptions around how certain types of ‘knowledge’ are defined as valued, and the hierarchy of value attached to different research paradigms which may disadvantage participatory research

•   The importance of interdisciplinary methods to understand the multiple influences creating patterns of health and wellbeing outcomes.

•   Drawing on sense making methods that work effectively with tacit knowledge, embodied experience, and emotions, to develop insights and knowledge.

•   Recognition of the importance of the values and purpose of research, including in support of social justice and broad human flourishing, rather than insistence on narrow notions of objectivity and instrumental rationality.

## Linking local understandings and action to broader action and social and policy change

In many ways PAR aims to nurture a consistent set of processes of collaborative inquiry and action across scales, to support equitable knowledge generation and social practice. Despite the ability of PAR to generate engagement and local innovation however, it is less often the case that broader action and social change is realised. While broader social change to address issues of social justice was an integral aim of the social movements that initially sought to promote participatory methods ^12^, the more circumscribed participation that forms part of development and research projects has often had more pragmatic, instrumental and sometimes extractive goals, fuelling recurring debates about the risk of co-optation noted above ^13^.

In the international development setting, participatory projects have sometimes generated motivation and ideas for change, only for local power structures and the wider social context to thwart potential wider action ^20, 95^. However, there are also examples of project funded PAR programmes that have been able to change entrenched social challenges, such as child labour or ethnic conflict ^41, 96^, rapidly share innovations through local learning networks ^97^, and explicitly work with wider networks for peacebuilding ^96^.

Scholars/practitioners have argued for the need for a longer-term approach than is typically supported in research or project funding cycles to sustain a participatory process over a period of 10 years or more, allowing insights and actions that emerge from the process to develop and address the systemic nature of many social challenges. Shorter-term projects may miss the opportunity to effectively link affected communities with a growth of organisational capacity to use the knowledge generated. It may also be difficult to capture these actions with traditional monitoring and evaluation frameworks. A growing body of participatory monitoring and evaluation draws attention to the quality of the inquiry process itself, as well as the need for addressing complexity and uneven trajectories of change over longer time frames ^98– 100^.

### Building on local knowledge and action

A case has been made that PAR is most appropriate for developing local knowledge and local action, with solutions rooted in nuanced understanding of the range of local factors that make a difference. It is in this context that the action learning cycle can most obviously be brought to bear, involving research, experience, analysis and action within a particular place or system ^3^. A question then arises: how is local insight and action connected to wider social change, particularly in addressing some of the structural drivers that may influence a locality but be generated and sustained beyond it. Given the ambitions of PAR to strive for broad real-world impact, proponents of PAR suggest that a broader understanding of generalisability may be needed. Methodologically, this may mean drawing on rigorous case studies and transferring partial explanations to new contexts – something for which realist approaches are particularly well suited. Practically, this may demand a learning infrastructure that links different initiatives and settings in an ongoing dialogue and exchange to share insights and adapt them locally ^97^ in what has been called an ‘association’ model of scale ^101^.

Argentinian and Brazilian scholars have developed a ‘genealogical approach’, drawing on Foucauldian insights around the mutual reinforcement of local meanings and practices and a bottom-up analysis of power, to suggest a need to build on local action and understanding for wider social justice initiatives ( 102 cited in 23). In other examples, PAR has fed into the action of trade unions and social movement organisations ^23^. In the related field of Asset Based Community Development, there is a similar emphasis on developing local insight and action first – both to build the most realistic picture of challenges that need addressing locally, but also to build the confidence and motivation to engage with wider systemic influences and other more powerful actors that may be impinging on the locality ^103, 104^.

The notion of an equity context ^105^ is useful for understanding whether the principles of valuing local experience and collaborative learning, sustained in a participatory approach, are echoed or undermined by the prevailing ways of working and wider social dynamics of organisations and agencies who are either funding, involved in, or expected to engage with participatory inquiry. A previous realist review of Community Based Participatory research highlights the importance of ‘partnership synergy’ and the quality of collaboration between different groups involved in research for research outcomes and the sustainability of relationships over time ^38^. Building on this work, a subsequent model makes visible important influences on successful CBPR including: social, historical and institutional context; the central role of partnership dynamics, with important ‘process’ outcomes including community empowerment, institutional capacity and policy changes ^106^. The challenges of linking and aligning diverse local actions and insights into broader coalitions for change is an area that would benefit from greater attention

### Vertical and horizontal contexts in health

In the case of health and health research, quite different contexts are provided by two broad approaches to health that can be characterized as ‘horizontal’ and ‘vertical’. In the 1970s, there was a recognition of the important role of the social determinants of health and well-being, and an attempt to develop a social model of health that was less focused on disease and medical interventions ^1, 107^. Comprehensive Primary Health Care (PHC) saw people’s involvement in deciding on priorities for action and capacity to act on a range of factors impinging on their everyday lives as an inherent component of health and wellbeing. These insights were reiterated with the extensive work done by the Commission for Social Determinants on Health decades later ^108, 109^. However, in the intervening period, a more ‘vertical’ approach was taken, emphasising programmes that specifically target key diseases such as Malaria, TB and HIV, or selective approaches to PHC. This alternative approach mobilized resources and research around medical treatments and infrastructure for specific health priorities, instead of focusing on systems-wide strengthening.

A vertical health paradigm that focuses on diseases, medicines and technology arguably remains dominant today, even while it is unevenly realised across diverse health systems, with the privatisation of health services and marketisation of health a dominant trend. At the same time, notions of the social determinants of health, and notions of social medicine and intercultural health more prevalent in LMICs, continue to assert the importance of a more systemic and interdisciplinary approach to wellbeing ^107^. Such approaches highlight the deficiencies of an emphasis on traditional economic growth and curative medicine and contribute to the emerging emphasis on ‘planetary health’ ^110^. The Covid-19 pandemic response has also highlighted how existing health inequalities can shape the pattern of health outcomes at the population level, and the important role of community driven responses for any effective and more equitable response ^111^. The distinct logic and relationships inherent in vertical and horizontal paradigms can present varied opportunities and constraints for a participatory approach to health and wellbeing that values the experience and insights of ordinary people and the heath promoting role of validating their knowledge and developing their capacity and agency.

In the previous section we highlighted how some environmental movements sought, in their actions and organising approaches, to prefigure the kinds of relationships and practices that are consistent with the future they are aiming to build. In this way, they show a recognition of how local action and practice needs to be consistent with the broader social relationships and type of society that is seen as desirable. This echoes the feminist adage that ‘the personal is political’ and the perennial concern of critical theory to understand the relationship between everyday practice and maintenance or challenge to the wider social regularities of which it is part. In the case of movements around mental health, a range of therapeutic communities from the late 1960s similarly sought to realise an alternative set of relationships and approaches that went against prevailing attitudes to mental health as well as psychiatric orthodoxy, with uneven results ^23^. Contemporary survivor and user-led movements highlight how apparent gains in ‘patient involvement’ over recent decades, are simultaneously undermined by the logic of austerity and punitive sanctions on disability benefits ^17, 112^.

In looking at participatory health research, the contexts in which it is attempted may thus have important implications for how far it is possible to be consistent with the paradigm as a whole.

### Understanding meanings, culture and social change

In understanding the limits and potential of PAR to contribute to wider social change, important insights may be drawn from broader literature on the dynamics of social change, and situating participatory practices and spaces within the wider flows of history and culture and more organic social change. Literature on social movements highlights how the ‘collective effervescence’ of group interaction, dialogue, and shared action can build a sense of belonging and develop new understandings and ‘framings’ of taken for granted social arrangements ^23, 113^. The generation of new understandings and mobilising symbols that galvanize people to act is an important creative dimension of social movement dynamics ^60^.

Recent theory of ‘community’, understood as a process rather than a thing, also provides important insights into how ‘beings and meanings in common’ are generated in different spaces of social interaction, while being simultaneously influenced by wider networks of resources and ideas ^114^. This is complemented by Social theory attempting to understand the broader dynamics of social change or lack of it ^33, 34, 37, 115, 116^ and the dynamic interplay between social practices, ideas and culture, networks of actors, and the influence of institutions and differential interests. Work under the rubric of ‘psychologies of liberation’ highlighted above, also emphasises the connections between prevailing social arrangements, the quality of inter-personal and group dynamics and individual psychology.

A strand of early critical theory has also sought to understand how communication, media, and cultural production play an important role in how some ideas and practices become institutionalized as an authorized ‘tradition’ or ‘culture’ ^117, 118^. As noted in the previous section, Escobar ^75^ has combined anthropologies of Latin American social movements and notions from design to reframe this as a question of actively designing ways of living and being, animated by distinctive principles of communality, care, and autonomy. From within the disability movement in the UK, Beresford frames this challenge as one that includes the construction of ideology, and notes that participation is rarely extended to shaping the overall ideology that organises and animates a society, including in some social movements which claim to advocate a democratic process ^18^.

### Influencing policy and practice

Work exploring the factors that support the uptake of research in policy and practice in health and development contexts has pointed to the importance of early and proactive engagement with a range of actors and networks to build opportunities for engaging with evidence ^119, 120^. In this context PAR is distinctive for the way that the research process itself tends to build relationships and networks for action, generating and supporting some of the links that promote uptake of research in policy and practice ^38^. A number of case examples where PAR has influenced policy change – such as in the case of HIV social movements – highlight the importance of better understanding conducive contexts and levers for change ^107^.

In separate debates around the anthropology of policy, there has also been a concern to understand how particular ideas and policy framings of issues emerge or are mobilised to organise disparate actors and initiatives at wider scales ^121, 122^. Such work draws on Foucauldian notions of discourse and the ‘dispositif’ and a bottom-up analysis of power. Similarly, the Deleuzian notion of ‘assemblages’ is also increasingly drawn on to understand the emergence of relatively enduring social institutions and patterns from heterogeneous practices, ideas, and material factors. This approach recognises the role of both powerful interests and the messy historical negotiations of social change ^123^.

### Section summary

In many ways, PAR seeks to intentionally democratise the character of social relationships and the process of knowledge production and action in a way that is consistent across scales The theoretical resources mentioned above may be helpful for understanding how the generative process of action-learning, supported by PAR, may or may not lead to influence on policy and practice in health and wider social change. It may also help analyse the following issues:

•   The process of generating local meanings and action from diverse perspectives and experiences

•   How understandings and actions can be linked across places and address structural factors that may be sustained beyond a locality.

•   The role of coalitions and networks in linking local action with wider organisations and systemic influences.

•   How local meanings and practices relate to prevailing culture, institutions, policies and ideology and either challenge them or are co-opted by them.

## Explaining Prticipatory Action Research

**Figure 2.**
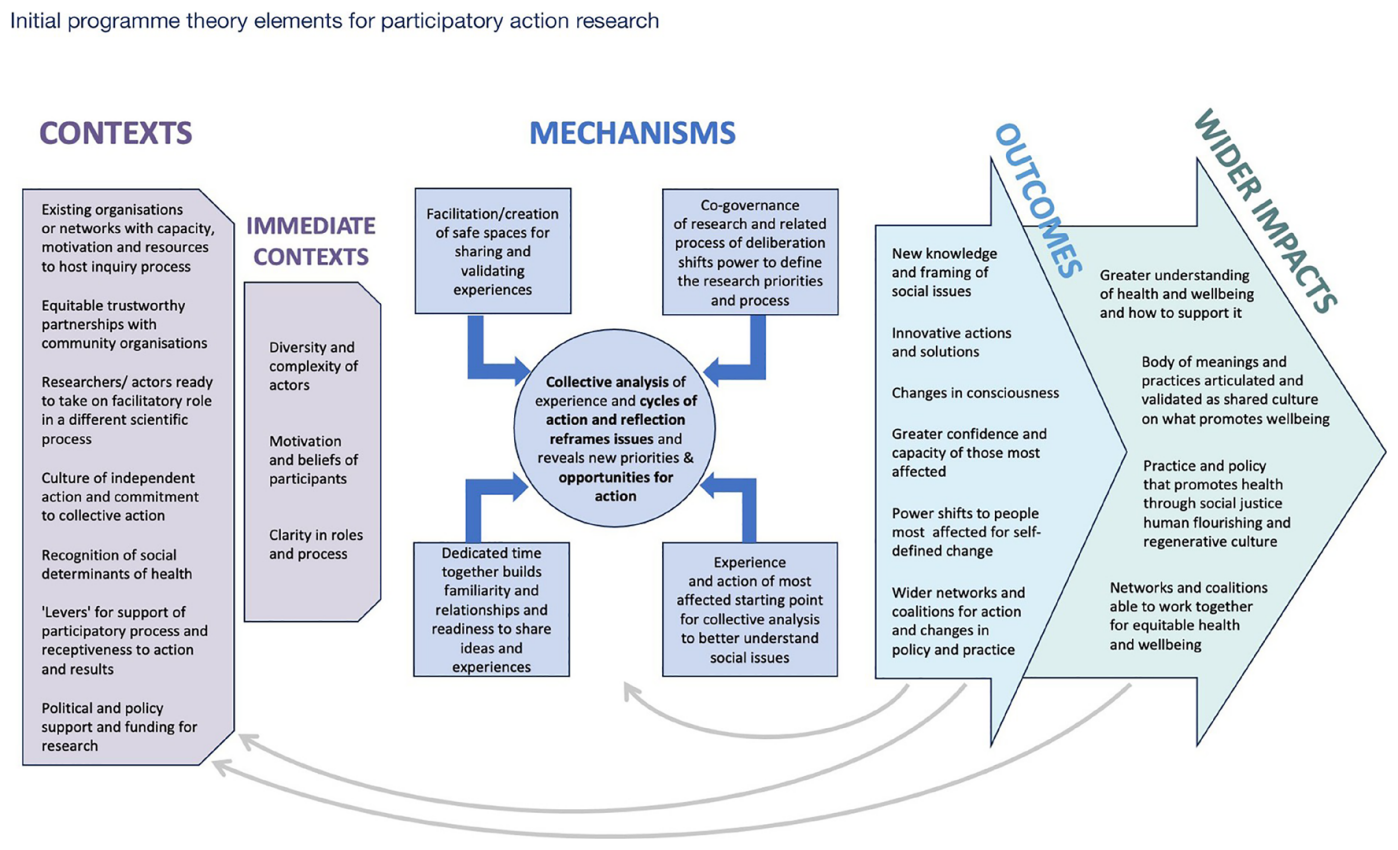
REAL2 initial Programme Theory for Participatory Action Research. Initial programme theory elements for participatory action research.

Drawing on the key issues explored above and the conceptual resources that help to understand the way in which they may play out in different contexts, we have developed a visualization that provides an initial explanatory account of PAR. Figure 2 illustrates how key relational mechanisms and influential contexts may lead to a range of outcomes - an initial ‘programme theory’ in realist terminology. This set of interlinked explanations sensitizes us to some of the key factors influencing PAR and will help us review the existing literature and further refine the programme theory in the light of available evidence as our REAL2 review progresses.

## Conclusions

In this paper we have outlined some important issues for further development of PAR as well as some conceptual resources that may help to deepen analysis around them. It is clear that many of the issues are interconnected, with relationships of power, conceptions of rigorous knowledge generation and links between local and wider action all mutually influencing each other. This initial mapping of issues and related theoretical resources will help us to construct the more systematic searches for a realist review of participatory research in health, and also help sensitise us to some of the connections during the analysis of the literature reviewed. In keeping with realist analysis, we will draw on some of the conceptual resources highlighted above, to construct plausible causal accounts of some of the key dynamics in play, in order to more systematically test them against literature in the field. Insights from practitioners/scholars and grey literature will also be important for the review in a field characterised by an emphasis on social action in the support of social justice, rather than solely on accounts of that process.

What is clear from our initial scoping, is that participatory action research implicates a wide range of social, group, psychological, epistemic, institutional and economic processes, all of which may need to be aligned for the process to realise it’s full potential and to avoid the process being co-opted and more limited Specifically, we aim to understand the factors that contribute to PAR being sustained as an iterative dialogue between action and knowledge generation in the pursuit of social justice and human and planetary wellbeing and flourishing.

## Ethics and consent statement

Ethics and consent were not required.

## Data Availability

No data are associated with this article
