# Supplementary material for: Mapping key issues and useful theory to address remaining ethical, practical and political challenges in participatory research in health
Source: Wellcome Open Res. 2025 Aug 4;10:412. [Version 1] doi: 10.12688/wellcomeopenres.24114.1 (PMC12640488; doi:10.12688/wellcomeopenres.24114.1)
Supplement: Peer review history

# Reviewer report: Reviewer response for version 1

**DOI:** 10.21956/wellcomeopenres.26603.r139157

**Published:** 2025-11-22

**Authors:** Corinna Elsenbroich

**Affiliations:** 1 University of Glasgow, Glasgow, Scotland, UK

This is really good review article for Participatory and Participatory Action Research. It motivates well why a review is needed. The realist stance is also well motivated. It discusses a range of approaches to PAR elicited from the review.

 One comment I have is that whilst the descriptive nature of the overall review is fine, I invite the authors to some more critical engagement with the broad range of perspectives and approaches in the conclusion. There is such a range of epistemological, ontological and methodological variations in the content of the paper that a better drawing together at the end would enhance the paper considerably.

Is the review written in accessible language?

Yes

Are all factual statements correct and adequately supported by citations?

Yes

Are the conclusions drawn appropriate in the context of the current research literature?

Yes

Is the topic of the review discussed comprehensively in the context of the current literature?

Yes

Reviewer Expertise:

participatory methods, health and health inequalities, complexity social science methods,

I confirm that I have read this submission and believe that I have an appropriate level of expertise to confirm that it is of an acceptable scientific standard.

# Reviewer report: Reviewer response for version 1

**DOI:** 10.21956/wellcomeopenres.26603.r129615

**Published:** 2025-10-03

**Authors:** Raphaela E. Kaisler

**Affiliations:** 1 Bertha von Suttner Private University St. Pölten GmbH, St. Pölten, Austria

Thank you for the opportunity to review this insightful and well-structured manuscript. The paper engages with important issues in Participatory Action Research (PAR), particularly challenges around power, knowledge generation, and the linking of local understandings and actions. These discussions are effectively connected to the development of a Program Theory of PAR. The model highlights the key elements of PAR and illustrates how these are influenced by context, ultimately shaping outcomes.

 I have a few minor points that I recommend the authors address:

- Power and control: Expand the discussion on further developments of Arnstein’s ladder (e.g., von Unger 2014; Wright 2020), including consideration of how Citizen Science can be situated within PAR.
- Page 6, first paragraph: Typo—“2060s” should likely read “1960s.”
- Page 6, third paragraph: Please elaborate on participatory tools and methods at specific stages of the research process, with illustrative examples.
- Reference 31: Please provide the complete reference.
- Page 7, group dynamics: Consider discussing the limited but growing body of empirical work on researcher–co-researcher dynamics and its facilitation in health research (e.g., McKenzie et al. 2016; Kaisler & Grill 2021; Ocloo et al. 2021).
- https://research-repository.uwa.edu.au/en/publications/barriers-to-community-involvement-in-health-and-medical-research-

Is the review written in accessible language?

Yes

Are all factual statements correct and adequately supported by citations?

Yes

Are the conclusions drawn appropriate in the context of the current research literature?

Yes

Is the topic of the review discussed comprehensively in the context of the current literature?

Yes

Reviewer Expertise:

I specialize in participatory processes in health and medical research, transdisciplinary research collaborations, as well as process and outcome research in psychotherapy and psychology, with a particular focus on empirical mixed-methods studies.

I confirm that I have read this submission and believe that I have an appropriate level of expertise to confirm that it is of an acceptable scientific standard.

## References

[1] : Enabling Transdisciplinary Collaboration: Stakeholder Views on Working With “Children With Mentally Ill Parents” Research Groups. *Frontiers in Psychiatry* .2021;12: 10.3389/fpsyt.2021.760716 10.3389/fpsyt.2021.760716 PMC864971534887787

[2] : Exploring the theory, barriers and enablers for patient and public involvement across health, social care and patient safety: a systematic review of reviews. *Health Research Policy and Systems* .2021;19(1) : 10.1186/s12961-020-00644-3 10.1186/s12961-020-00644-3 33472647 PMC7816359
